# Fat Mass Centile Charts for Brazilian Children and Adolescents and the Identification of the Roles of Socioeconomic Status and Physical Fitness on Fat Mass Development

**DOI:** 10.3390/ijerph13020151

**Published:** 2016-01-22

**Authors:** Simonete Silva, Adam Baxter-Jones, José Maia

**Affiliations:** 1 Department of Physical Education, University Regional of Cariri, Ceará 63112-012, Brazil; simonete.silva@urca.br; 2 College of Kinesiology, University of Saskatchewan, Saskatoon, SK S7N 5B2, Canada; baxter.jones@usask.ca; 3 Centro de Investigação, Formação, Intervenção e Inovação em Desporto (CIFI^2^D), Kinanthropometry Lab, Faculty of Sport, University of Porto, Porto 4200-450, Portugal

**Keywords:** reference percentile curves, fat mass, changes, children, adolescents

## Abstract

This paper presents fat mass centile charts for Brazilian youth and investigates the roles of socioeconomic status and physical fitness (PF) on fat mass (FM) development. Two northeast Brazilian samples were used: a cross-sectional sample of 3659 (1921 girls) aged 8 to 16 years and a mixed-longitudinal series of cohorts (8–10, 10–12, 12–14, 14–16 years) with 250 boys and 250 girls. A measure of somatic maturity was used as a marker of biological maturation; PF comprised agility, explosive and static strength, and aerobic capacity. Socioeconomic status was based on school attended; public or private. Slaughter’s anthropometric equations were used to estimate FM. Percentile charts was constructed using the LMS method. HLM (Hierarchical Linear Model) 7 software modeled FM changes, identifying inter-individual differences and their covariates. Girls and boys had different FM percentile values at each age; FM increased nonlinearly in both girls and boys. Higher PF levels reduced FM changes across time in both sexes. Sex-specific non-linear FM references were provided representing important tools for nutritionists, pediatriciann and educators. Physical fitness levels were found to act as a protective factor in FM increases. As such, we emphasize PF importance as a putative health marker and highlight the need for its systematic development across the school years.

## 1. Introduction

Anthropometric markers of physical growth and body composition (BC) are considered important health indicators at both the individual and population levels [1]. Further, growth reference values, expressed via percentile charts, are among the most widely used, and important instruments, in public health and clinical settings [1,2,3]. They are also used by nutritionists, physical educators and pediatric sport scientists [4,5,6]. It is recommended that reference values should be from the population under study, as cultural and ethnic differences affect a child’s growth and development.

The prevalence of youth obesity worldwide has increased dramatically [7]. Additionally, this trend is also linked to low physical activity levels as well as to insufficient health-related physical fitness which apparently contribute to the development of type 2 diabetes, metabolic syndrome, cardiovascular diseases, and all-cause mortality in both adults and children [8,9]. It is thus important to closely monitor fat mass development in children and youth, and the need for country specific reference values is of the utmost relevance. There are currently available several models and techniques to quantify fat mass, varying in complexity and ease of use [10]. When data is gathered in the community on hundreds of subjects, skinfold measurements are amongst the cheapest markers to help in assessing and estimating fat and fat free mass development [10] across age groups in a variety of cultural contexts [11]. Equations to estimate fat mass from skinfolds have been derived and used in many developed [12] and developing countries [11]. Percentile reference charts for fat mass have been developed for children and adolescents for use in US populations [13], as well as for Mexicans’ [14], Germans’ [15] and Turks’ [16], to name but a few. However, the ability of these cross-sectional developed charts to precisely illustrate children and adolescents growth is compromised as they do not capture the longitudinal change patterns seen within and between sexes that occur at different times and rates [17]. It is well known that BC and physical fitness (PF) parameters show variation in their development with age and biological maturation [18]. There is also considerable evidence that low socioeconomic status (SES) is associated with an increased risk of obesity in children [19]. The search for meaningful relationships between PF levels, SES, biological maturation and BC development is an important and challenging endeavor because of their links with children and adolescents health [20].

Although longitudinal data showing the influence of PF, physical activity (PA) and biological maturation on fat mass changes during childhood and adolescence is scarce [21], cross-sectional data report that moderate-to-high levels of PF and PA have an inverse relationships with overweight/obesity in children and youth [22,23,24,25]. Further, available epidemiological longitudinal data mostly use BMI (Body Mass Index) as a putative marker of body fat (BF) changes. For example, Martins *et al.* [26] using Azorean child data, followed consecutively from 6 to 10 years, did not find any significant associations of PA and PF on their annual BMI changes. Conversely, De Souza *et al.* [27] reported that higher PF levels reduced BMI changes across time in Portuguese youth, followed from 10 to 18 years. Additionally, Byrd-Williams *et al.* [28] identified that greater cardiorespiratory fitness was protective against increasing adiposity in Hispanics boys, but that in Hispanic girls no such significant associations were found. Although BMI is widely used as a proxy for body fat, given its measurement simplicity and valuable use in monitoring obesity trends in population studies, it does have some limitations. The major disadvantage of using BMI is that it does not distinguish between tissue types. Thus increased BMI can result from both increased fat mass and/or increased lean mass. This can lead to significant misclassification of obesity status [29].

This paper aims to develop longitudinal fat mass centile charts for Brazilian children and investigate the roles, if any, of socioeconomic status and physical fitness on fat mass development. It is hypothesized that: (1) Brazilian boys and girls fat mass reference centile charts will show clear sex-differences; (2) fat mass longitudinal changes will show different trends in boys and girls; (3) socioeconomic status will act as a moderator covariate; (4) higher levels of PF will act as protective forces in the reduction of BF increases; and (5) that advanced biological maturation and high PF performance will reduce fat mass development.

## 2. Methods

### 2.1. Participants

Data are taken from the “Healthy Growth in Cariri” project. This study comprises two distinct and representative samples of randomly selected children and adolescents from public and private schools simultaneously studied from 2006 to 2009 [6,30]. The first sample comes from a mixed-longitudinal study comprising groups of individuals aged 8 to 16 years, from four baseline age cohorts (8, 10, 12, and 14 years) with overlapping years as shown in Table 1. Individuals were measured at 6 month time intervals. The study aim was to investigate change and stability of growth, body composition, and motor performances (Table 1). The second sample originated from a cross-sectional study (Table 2). All subjects came from three cities in the Cariri region: Juazeiro do Norte, Crato, and Barbalha located in Ceará State, Northeast of Brazil. Both samples comprised healthy children and youth with parental permission to participate in the study. Participants were recruited by invitation after receiving school principals’ permission to be contacted to participate in the study. The response rate for participation was ~100%. 

**Table 1:** Mixed-longitudinal sample size by age, cohorts and sex (number of observations).

Cohort	Ages (years)	Girls	Boys	Total
C1	(8–9–10)	690	834	1524
C2	(10–11–12)	570	516	1086
C3	(12–13–14)	606	678	1284
C4	(14–15–16)	252	324	576
	Total	2118	2352	4470

**Table 2:** Cross sectional sample size by age and sex (number of observations).

Ages	Girls	Boys	Total
8	115	132	247
9	136	154	290
10	160	131	291
11	224	192	416
12	309	288	597
13	371	287	658
14	267	239	506
15	207	185	392
16	132	130	262
Total	1921	1738	3659

### 2.2. Ethical Statement

All subjects gave their informed consent for inclusion before they participated in the study. The *“Healthy Growth in Cariri”* was conducted in accordance with the Resolution N° 466 of National Health Council, Brazil, and the protocol was approved by the Ethics Committee of the Medical School of Juazeiro do Norte, CE, Brazil (N° 01/07).

### 2.3. Dependent Variables

#### Anthropometrics

All measurements were made by trained staff following the same procedures as given by Claessens *et al.* [31]. Height and sitting height were measured using a portable stadiometer with head positioned to the Frankfurt plane (CARDIOMED^®^ Welmy Model 220, Curitiba, Paraná, Brazil); sitting height was measured with subjects sitting on a bench of known height (40 cm). Leg length was calculated as the difference between height and sitting height. Body mass was measured using a digital scale (TANITA^®^ Model 683W, Taiwan, China) and skinfold thickness (tricipital and subscapular) were measured as a double fold of skin underlying the soft tissue on the left side of the body using Holtain calipers. Fat mass was estimated from these skinfold measures using Slaughter sex-specific equations [32], which are widely used in Brazil [33] as well as in other cultural contexts [13,34,35], and also because it shows valid results when contrasted to DXA (Dual-energy X-ray Absorptiometry) measurements [36].

### 2.4. Time-Invariant Predictor

#### Socioeconomic Status

Socioeconomic status was defined according to which school children attended: private (high level) and public (low level).

### 2.5. Time-Varying Predictors

#### Maturity Status

Decimal age was calculated from the birth date to the date of measurement; categorical age (CA) was organized in groups (7.49–8.50 = 8 years). A biological age (a measure of maturity) was estimated by identifying how far an individual was from attaining or having attained peak height velocity (PHV). In brief, sex-specific anthropometric equations were used to predict the age when PHV would be attained; covariates used included: CA, body mass, height, sitting height and leg length [37]. PHV is a comparable sexual milestone found in both boys and girls; the time from the attainment of PHV is coined as the maturity offset; at PHV maturity offset = 0. The maturity offset equation estimates the distance each subject is from their expected age (in years) of attainment of PHV. The values are expressed in years (either + or −) from PHV.

### 2.6. Physical Fitness

Performance-related PF was assessed using four tests, from two widely used batteries: The AAHPERD [38] and the EUROFIT [39] which include measures of cardio respiratory endurance (a 12-min run), agility/speed (shuttle-run 10 × 5 m), static strength (hand grip), and power (standing long jump):

The 12-min run test: in a previously delimited field, schoolchildren in groups of 10–12 (male, female) run/walk the maximum possible distance in 12 min.

Standing long jump was measured as the distance from the take-off line to heel or other part of the body that touched the floor nearest the take-off line. Children stood with feet apart behind the take-off line and were instructed to jump as far as possible. Two trials were recorded and the better of the two trials was retained for analysis.

The grip-strength test: the subjects were instructed to squeeze a calibrated hand dynamometer (Takei TKK 5401, Tokyo, Japan) with maximal force. All schoolchildren performed two trials with each hand. The best trial from each hand was recorded in kg and was used to compute the mean muscle strength. The handle length was adjusted to control for variations in hand size.

The shuttle-run test: each subject performs five cycles (round-trip) at maximum speed between two lines separated by five meters (total distance = 50 m); this test was conducted in pairs, and the best trial was used in the analysis.

Approximately 30 to 40 children were assessed on a daily basis in all tests according to a standardized station setting. The cardiorespiratory test (12 min-run) was always administrated at the end of the data collection day. This procedure was tested in the pilot study after careful decision about the best testing sequence.

### 2.7. Data Quality Control

Data quality control was done in two steps. Team members were trained in and applied all testing procedures on a sample of 26 children (pilot study), and an in-field reliability procedure was implemented during the data collection. In each assessing day, a random sample of five children was retested. Intra-observer technical error of measurement (TEM) and ANOVA-based intraclass correlation coefficients (R) (test-retest) were computed; the obtained values were as follows: triceps and subscapular skinfolds TEM = 0.5 mm and 1.0 mm, respectively; further, R varied from 0.87 (95%CI—Confidence Interval = 0.72–0.94) in the 12-min run to 0.96 (95%CI = 0.95–0.97) in the standing long jump.

### 2.8. Statistical Analyses

#### 2.8.1. Centile Charts

Descriptive statistics were computed using IBM-SPSS 20 software (IBM Corporation, New York, NY, USA). Centile curves for fat mass were constructed separately for each sex using the LMS method [40,41]. A Box-Cox power transformation was used to normalize the data at each age. Natural cubic splines with knots at each distinct age (*t*) were fitted by maximum penalized likelihood to create three smooth curves: *L(t)* the Box-Cox power, *M(t)* the median and *S(t)* the coefficient of variation. Centile curves at age *t* were then obtained as:

$$\left[C_{100a}(t)=M(t){\left[1+L(t)S(t)Z_{a}\right]}^{1/L(t)}\right]$$

where *Z*_α_ is the normal equivalent deviate for tail area α and *C_100_*_α_*(t)* is the centile corresponding to *Z*_α_. Equivalent degrees of freedom (edf) for *L*(*t*), *M*(*t*) and *S*(*t*) measure the complexity of each fitted curve. Q tests [42,43] were used to check the goodness of fit with the aid of deviance measure of penalized likelihood [41,42,44]. These analyses were made in lmsChartmaker software [45].

#### 2.8.2. Multilevel Modelling

All analyses were stratified by sex. No moderate or severe outliers (beyond one and a half box lengths in the box-plot representations), nor leverage points were identified. Modelling longitudinal changes in fat mass was performed using the HLM 7.01 software (Scientific Software International, Inc, Shokie, IL, USA). All parameters were simultaneously estimated by maximum likelihood techniques with robust standard-errors [46]. In HLM analysis, the numbers and spacing of measurement observations may vary across subjects and the analysis can also accommodate data from mixed-longitudinal designs with missing data, under the assumption that data is missing is at random [47]. This is a very realistic assumption in longitudinal studies, and the mixed-effects regression models are highly efficient in terms of parameter estimates [48]. A series of hierarchical nested models was fitted to the data using a “stepwise” approach. To facilitate interpretation of model parameters describing fat mass changes, the time metric was centered at baseline, *i.e.*, the first measurement of cohort 1, *i.e.*, 8 years. Model 1 (M_1_) was fitted to identify the best descriptors of individual fat mass trajectories using polynomials of age (up to a 3rd degree, *i.e.*, age, age^2^, and age^3^). In model 2 (M_2_), SES was added as a fixed covariate. In the final model (M_3_), time-varying covariates, namely biological age, shuttle run time, long jump length, 12 min run distance and hand grip strength (expressed as relative strength: strength/body mass) were added. A deviance statistic was used as a measure of global fit. Differences in deviances are distributed as an approximate Chi-square (χ^2^) distribution with degrees of freedom equal to the difference in the number of estimated parameters between two nested models. As models’ increase in complexity, *i.e.*, with more parameters, statistical significant decrease in deviance is expected due to a better fit of the data. All time-varying predictors were centered to their grand means.

## 3. Results

Descriptive statistics for girls and boys at each time point (8 to 16 years of age) are shown in Table 3. As expected, boys and girls average heights, weights and fat masses increased until 16 years of age. On average, girls’ estimated age at PHV was ~13 years, while in boys it was ~15 years. Boys showed systematic average improvements in all PF tests. Girls showed average increments in strength (explosive leg power and hand grip) with age, compared to no changes in their 12-min run or shuttle-run performances after 10 years of ages.

**Figure 1:**
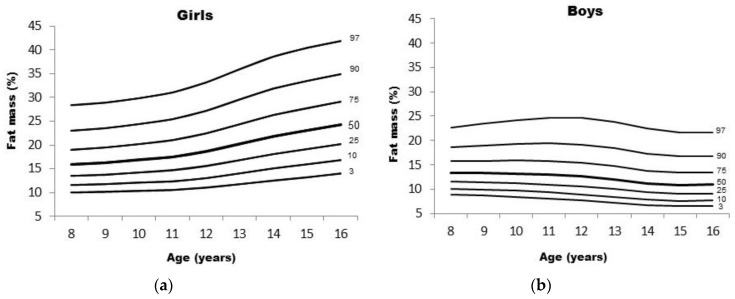
Centile curves for body mass of girls (**a**) and boys (**b**).

Table 4 shows numerical values of the data in the channels round the 7 fitted centiles of 3rd, 10th, 25th, 50th, 75th, 90th, and 97th of fat mass. Figure 1 shows reference curves of fat mass for children of both sexes. These curves show different shapes in boys and girls. Girls’ fat mass 50th percentile was higher than boys and increase almost linearly from 8 to 16 years, while in boys it increased until 11 years; from 12 years onwards it gradually decreased. 

The results of the multilevel models are shown in Table 5 (girls) and Table 6 (boys). In girls, the best model describing intra-individual fat mass changes had a non-linear trend (3rd degree polynomial), with *p*-values very close to 0.05. Model 1 can be interpreted to mean that, on average, fat mass of an 8 years old girl was 16.7%, followed by positive (velocity and acceleration) and mean negative curvatures (age^3^). While Model 2 maintains the curve shape, the average fat mass drops to 15.9%, for an 8 years old girl when SES is accounted for. Thus at 8 years of age those from private schools had more fat mass (+3.0%). No significant effects of SES were found for % fat mass changes across any other ages. Model 2 fitted the data better, indicated by a change in deviance (*i.e.*, 6227.143(M_1_) − 6201.162(M_2_) = 25.981 with 4 df, *p* < 0.001). In Model 3 SES effects were dropped; this proved to be the best fitting model of the three (*i.e.*, deviance 6201.162(M_2_) − 4209.196(M_3_) = 1991.966 with 2 df, *p* < 0.001). In Model 3, the average fat mass of a girl from a public school experiencing her PHV, when holding all other predictor variables at their respective means, was 24.7%. Those from private schools had 1.9% more fat mass when the other confounders were held constant. Time-varying covariates (shuttle-run) did not independently predict (*p* = 0.403) fat mass development with age. Those advanced in their biological maturation had more fat mass (β = 3.265 ± 0.320), but those with more explosive (SLJump, β = −0.026 ± 0.080) and relative static strength (Hand-grip, β = −9.308 ± 1.829), as well as higher aerobic fitness (12’ run, β = −0.010 ± 0.005) showed lower fat mass changes.

Boys’ fat mass trajectories in Model 1 showed that, on average, at 8 years of age fat mass was 14.5%. A non-linear (curvature) change in fat mass was evident, since age (β = 1.468 ± 0.364), age^2^ (β = −0.481 ± 0.118), and age^3^ (β = 0.031 ± 0.010) parameter estimates were all statistically significant (*p* ≤ 0.003). In Model 2, SES effects were only significant at baseline, indicating that boys from private schools had, on average, at 8 years of age 6.2% more fat mass than their public school peers. Model 2 fitted the data better than Model 1 [deviance is 6074.389(M_1_) − 5992.822(M_2_) = 81.567 with 4 df, *p* < 0.001]. In Model 3, the best model (deviance 5992.822(M_2_) − 4158.121(M3) = 1834.701 with 2 df, *p* < 0.001), the non-significant SES effects was discarded and biological age and PF components were added as time-varying predictors of changes in fat mass. The average fat mass of a boy from a public school experiencing his PHV was 15.9%, when all other time-varying predictors were held constant to their respective means. Those from private schools had, on average, 4.5% more fat. Mean velocity (β = 1.536 ± 0.606), acceleration (β = −0.448 ± 0.158), and a cubic (β = 0.025 ± 0.013) trend was consistenly evident in fat changes, mirroiring the picture observed in their centile charts. The more mature boys had higher fat mass values (β = 1.089 ± 0.260), whereas the fittest, *i.e.*, with more explosive (β = −0.053 ± 0.010) and relative static strength (β = −5.974 ± 0.185), as well as aerobic fitness (β = −0.007 ± 0.003) showed lower fat mass changes.

**Table 3:** Descriptive statistics for girls and boys in each annual time point (8 to 16 years of age).

Variables		8 Years	9 Years	10 Years	11 Years	12 Years	13 Years	14 Years	15 Years	16 Years
		(*n*) Mean ± SD	(*n*) Mean ± SD	(*n*) Mean ± SD	(*n*) Mean ± SD	(*n*) Mean ± SD	(*n*) Mean ± SD	(*n*) Mean ± SD	(*n*) Mean ± SD	(*n*) Mean ± SD
*Anthropometrics measures*										
Heigth (cm)	Girls	(67) 125.2 ± 6.0	(139) 127.2 ± 6.2	(166) 135.5 ± 7.3	(179) 139.6 ± 7.7	(161) 146.8 ± 8.1	(179) 150.0 ± 7.7	(136) 154.1 ± 6.6	(110) 156.2 ± 6.2	(56) 157.6 ± 6.1
	Boys	(75) 125.1 ± 6.1	(153) 128.5 ± 6.9	(196) 134.6 ± 6.9	(177) 137.6 ± 6.6	(141) 144.5 ± 6.9	(191) 149.1 ± 7.6	(179) 156.9 ± 7.3	(131) 160.4 ± 7.4	(55) 166.7 ± 5.6
Weight (kg)	Girls	(67) 24.2 ± 4.4	(139) 27.3 ± 6.3	(166) 33.3 ± 9.6	(179) 35.9 ± 9.6	(161) 41.3 ± 10.6	(179) 45.1 ± 10.9	(136) 47.5 ± 10.9	(110) 48.9 ± .0	(56) 50.5 ± 5.8
	Boys	(75) 26.6 ± 6.3	(153) 28.5 ± 7.7	(196) 32.5 ± 8.4	(177) 34.0 ± 8.3	(141) 37.8 ± 8.2	(191) 42.0 ± 8.9	(179) 47.5 ± 8.9	(131) 50.5 ± 8.0	(55) 57.9 ± 9.5
Fat mass (%)	Girls	(67) 16.8 ± 5.1	(128) 16.4 ± 4.3	(147) 18.9 ± 6.0	(157) 18.6 ± 5.9	(155) 21.6 ± 8.2	(174) 23.3 ± 8.9	(134) 24.0 ± 7.6	(109) 23.9 ± 7.9	(55) 24.7 ± 5.5
	Boys	(69) 15.2 ± 5.3	(137) 14.3 ± 4.7	(161) 15.3 ± 5.6	(155) 13.4 ± 5.0	(132) 14.5 ± 6.1	(178) 14.4 ± 5.8	(159) 10.8 ± 3.5	(122) 11.3 ± 3.9	(51) 13.6 ± 5.8
Maturity Offset (years)	Girls	(67) −3.51 ± 0.35	(139) −2.97 ± 0.48	(166) −1.99 ± 0.57	(179) −1.36 ± 0.60	(161) −0.35 ± 0.62	(179) 0.29 ± 0.57	(136) 1.14 ± 0.51	(110) 1.68 ± 0.49	(56) 2.49 ± 0.46
	Boys	(75) −4.61 ± 0.34	(152) −4.25 ± 0.48	(196) −3.51 ± 0.53	(178) −3.15 ± 0.47	(141) −2.27 ± 0.56	(191) −1.72 ± 0.59	(179) −0.70 ± 0.68	(131) −0.08 ± 0.70	(55) 1.11 ± 0.50
*Physical Fitness tests*										
12-minute run (m)	Girls	(54) 1431.6 ± 126.6	(139) 1570.4 ± 348.2	(117) 1601.8 ± 280.1	(151) 1647.2 ± 258.3	(148) 1641.7 ± 283.0	(164) 1632.8 ± 321.1	(123) 1680.6 ± 326.9	(87) 1624.9 ± 339.1	(40) 1619.3 ± 250.8
	Boys	(57) 1445.6 ± 173.8	(153) 1593.3 ± 369.9	(152) 1764.0 ± 371.1	(156) 1827.0 ± 353.8	(137) 1884.5 ± 342.4	(180) 1989.4 ± 390.1	(168) 2116.0 ± 334.6	(124) 2165.4 ± 380.8	(47) 2161.9 ± 364.5
SLJ (cm)	Girls	(67) 109.6 ± 22.2	(139) 121.4 ± 21.6	(165) 125.9 ± 22.0	(173) 130.6 ± 23.0	(159) 134.5 ± 20.4	(177) 139.9 ± 21.9	(131) 139.5 ± 19.6	(107) 138.7 ± 22.6	(51) 137.4 ± 17.4
	Boys	(57) 117.0 ± 22.0	(153) 128.7 ± 20.6	(195) 136.2 ± 18.7	(176) 142.7 ± 18.5	(141) 146.4 ± 19.5	(188) 158.2 ± 21.2	(174) 174.4 ± 23.4	(129) 178.9 ± 25.4	(55) 178.3 ± 26.8
SHR (s)	Girls	(67) 25.7 ± 1.9	(130) 24.5 ± 2.2	(164) 23.7 ± 1.8	(171) 23.2 ± 1.8	(158) 22.8 ± 1.7	(171) 22.6 ± 1.6	(129) 22.8 ± 1.9	(100) 22.9 ± 2.0	(49) 23.2 ± 1.9
	Boys	(57) 24.7 ± 2.2	(153) 23.3 ± 1.8	(191) 22.4 ± 3.7	(174) 22.1 ± 1.5	(140) 22.0 ± 1.6	(185) 21.4 ± 1.7	(171) 20.7 ± 1.6	(130) 20.5 ± 1.6	(50) 20.5 ± 1.7
HGr (kg)	Girls	(67) 9.5 ± 2.0	(139) 10.6 ± 2.6	(158) 14.7 ± 3.7	(161) 15.9 ± 4.2	(144) 18.8 ± 5.7	(164) 20.8 ± 5.3	(120) 22.5 ± 5.5	(102) 24.1 ± 4.4	(55) 26.6 ± 3.4
	Boys	(57) 11.0 ± 2.9	(153) 11.7 ± 2.7	(180) 15.2 ± 3.8	(158) 16.2 ± 3.8	(122) 19.5 ± 4.5	(167) 22.5 ± 5.4	(154) 28.7 ± 7.2	(109) 33.2 ± 7.1	(52) 36.0 ± 8.0

**Table 4:** Numerical values for centile fat mass curves by sex and age group.

Girls—Fat Mass (%)										Boys—Fat Mass (%)								
Ages	L	S	3rd	10th	25th	50th	75th	90th	97th	L	S	3rd	10th	25th	50th	75th	90th	97th
8	−0.398	0.257	9.98	11.55	13.50	15.94	19.04	23.05	28.36	−0.557	0.228	8.92	10.11	11.58	13.40	15.71	18.70	22.69
9	−0.344	0.260	10.11	11.75	13.78	16.30	19.49	23.57	28.89	−0.546	0.243	8.64	9.87	11.39	13.30	15.77	19.01	23.42
10	−0.286	0.264	10.32	12.06	14.20	16.86	20.19	24.42	29.86	−0.536	0.258	8.38	9.65	11.22	13.23	15.85	19.37	24.23
11	−0.227	0.269	10.52	12.38	14.66	17.49	21.01	25.43	31.07	−0.525	0.272	8.05	9.32	10.92	12.99	15.73	19.44	24.69
12	−0.169	0.275	11.00	13.04	15.54	18.62	22.43	27.19	33.17	−0.514	0.283	7.67	8.93	10.52	12.60	15.37	19.17	24.59
13	−0.115	0.279	11.76	14.03	16.80	20.20	24.39	29.56	35.99	−0.503	0.290	7.22	8.43	9.98	12.00	14.71	18.44	23.80
14	−0.064	0.281	12.55	15.06	18.10	21.80	26.32	31.84	38.63	−0.492	0.293	6.72	7.86	9.32	11.23	13.79	17.33	22.41
15	−0.019	0.279	13.24	15.92	19.16	23.07	27.79	33.50	40.41	−0.481	0.293	6.50	7.62	9.04	10.89	13.37	16.78	21.66
16	0.025	0.275	13.96	16.80	20.20	24.26	29.13	34.94	41.87	−0.471	0.291	6.57	7.69	9.12	10.98	13.46	16.85	21.67

**Table 5:** Results summary for the three nested models of girls.

Variables	Model 1		Model 2		Model 3	
	Estimate (SE)	*p*-Value	Estimate (SE)	*p*-Value	Estimate (SE)	*p*-Value
*Fixed effects*	–	–	–	–	–	–
Intercept	16.709 (0.547)	<0.001	15.919 (0.649)	<0.001	24.719 (1.414)	<0.001
SES	–	–	3.003 (1.169)	0.011	1.848 (0.815)	0.024
Age	0.641 (0.352)	0.069	0.275 (0.421)	0.515	−0.333 (0.566)	0.557
SES	–	–	1.276 (0.758)	0.093	–	–
Age^2^	0.199 (0.107)	0.062	0.300 (0.126)	0.018	−0.018 (0.149)	0.511
SES	–	–	−0.349 (0.233)	0.134	–	–
Age^3^	−0.017 (0.009)	0.053	−0.023 (0.011)	0.032	0.007 (0.012)	0.549
SES	–	–	0.018 (0.019)	0.366	–	–
Maturity offset	–	–	–	–	3.265 (0.320)	<0.001
Shuttle-run (s)	–	–	–	–	0.063 (0.075)	0.403
SLJump (m)	–	–	–	–	−0.026 (0.080)	0.002
12‘ run (m∙m^−1^)	–	–	–	–	−0.010 (0.005)	0.040
Hand-grip (kg∙kg^−1^)	–	–	–	–	−9.308 (1.829)	<0.001
*Random effects*	–	–	–	–		–
Intercept	44.085	<0.001	41.826	<0.001	33.067	<0.001
Residual	5.572	–	5.512	–	5.623	–
*Deviance*	6227.143	–	6201.162	–	4209.196	–

**Table 6:** Results summary for the three nested models of boys.

Variables	Model 1		Model 2		Model 3	
	Estimate (SE)	*p*-Value	Estimate (SE)	*p*-Value	Estimate (SE)	*p*-Value
*Fixed effects*	–	–	–	–	–	–
Intercept	14.531 (0.419)	<0.001	13.234 (0.379)	<0.001	15.860 (1.372)	<0.001
SES	–	–	6.145 (1.176)	<0.001	4.572 (0.771)	<0.001
Age	1.468 (0.364)	<0.001	1.209 (0.335)	<0.001	1.536 (0.606)	0.012
SES	–	–	1.423 (1.095)	0.192	–-	–
Age^2^	−0.481 (0.118)	<0.001	−0.390 (0.106)	<0.001	−0.448 (0.158)	0.005
SES	–	–	−0.576 (0.390)	0.141	–	–
Age^3^	0.031 (0.010)	0.003	0.025 (0.009)	0.006	0.025 (0.013)	0.050
SES	–	–	0.043 (0.035)	0.219	–	–
Maturity offset	–	–	–	–	1.089 (0.260)	<0.001
Shuttle-run (s)	–	–	–	–	0.108 (0.071)	0.126
SLJump (m)	–	–	–	–	−0.225 (0.007)	<0.001
12‘ run (m∙m^−1^)	–	–	–	–	−0.007 (0.003)	0.033
Hand-grip (kg∙kg^−1^)	–	–	–	–	−5.974 (0.185)	0.001
*Random effects*	–	–	–	–	–	–
Intercept	28.947	<0.001	24.065	<0.001	22.680	<0.001
Residual	4.113	–	4.032	–	3.958	–
*Deviance*	6074.389	–	5992.822	–	4158.121	–

## 4. Discussion

This study present centile charts and reference fat mass values for Cariri (Brazilian) children and adolescents. These are very important tools for monitoring fat mass growth patterns when interpreting their annual increments. Additionally, we also show a series of multilevel models, sequentially fitted, in order to investigate fat mass longitudinal changes as well as their associations with subjects’ PF levels, sex, biological maturation, and SES status.

Fat mass centile charts display different patterns between males and females. In girls, fat mass 50th percentile increased almost linearly; an average 8 years old girl fat mass was 15.94%, by 16 years of age it increased to 24.26%, representing an increase of 8.32%. In contrast, boys’ fat mass centiles showed systematic decreases with age; at 8 years of age the 50th percentile (P_50_) fat mass was 13.40%, by 16 years of age the P_50_ was 10.98%, *i.e.*, a 2.5% reduction. Similar fat mass centile shapes were presented in other studies, but their values were relatively higher. For example, US children and adolescents’ data reported by Laurson, Eisenmann and Welk [13] showed that an 8 year old girl’s fat mass was 17.9% and a boy’s was 15.5%. Similarly, reported data from Cicek *et al.* [16] of Turkish children (6–17 years), based on four skinfolds, also showed that fat mass was 18.1% and 17.6% at 8 years of age, for girls and boys respectively. Furthermore, and based on bio-impedance data from English children and adolescents (5–18 years), fat mass reports from McCarthy *et al.* [49] showed that 8 year old girls’ 50th percentile was 24.1%, and in boys it was 19.5%; a similar trend was also seen in reference values reported by Plachta-Danielzik *et al.* [15] with German children and adolescents (3–16 years). These consistent different values seen across centiles questions the validity of a “universal reference”, this was clearly pointed out by Olds [12] and relates to the fact that fat mass values in adolescents are the net results of the complexities of growth and development processes during puberty. Further, socioeconomic levels and ethnicity are also confounding factors that should be taken into account when comparisions between populations are made. On the other hand, and although there is an abundance of percentile BMI data, it is well-known that BMI values are expressed in an imperfect metric of fatness, and do not characterize fat distribution [50]. Additionally, skinfolds accurately and reliably measured by trained anthropometrists provide much better information about body composition changes, which may be obscured using BMI. For example, the combination of dieting and lack of exercise may result in some adolescent girls having relatively low BMIs but high levels of body fat, as suggested by Olds [12].

Given the marginally differences in intercepts and non-linear trends among models (M_1_ to M_2_) of fat mass development, the present discussion will focus on the best fitting models (M_3_). Physical fitness tests, as time-varying predictors, were negatively related to fat mass’ trajectories, suggesting that fitter children and adolescents (estimated from standing long jumps, 12-min runs and hand grip strengths) had lower mean fat mass changes. This relation is particularly marked in tests in which the body is projected or moved through space (jumping and running/endurance and velocity), because fat represents an excess of weight that has to be moved during these tasks, as well as the lack of experience in such weight-bearing tests [18,25]. This is relevant information indicating that consistent PF improvements, namely in cardiorespiratory fitness, speed, and strength, during late childhood and adolescence may play an important roles in reducing fat mass accumulation. Although several studies [24,51,52] have examined the independent associations of PF with fat mass, very few have examined longitudinal fat mass changes using the multilevel modelling approach used in the present study. De Souza *et al.* [27] using such a multilevel approach to model changes in BMI revealed, in a mixed-longitudinal study with 6.894 Portuguese adolescents, that BMI increased almost linearly with age and that those with better PF levels had lower mean BMI changes; these results are consistent with the trend found in the present study.

Socioeconomic status was found to be related to fat mass only at 8 years of age, *i.e.*, those children and adolescents from private schools had greater fat mass than those from the public schools. This may be linked to their greater access to video-games/computer use and thus a predicted increase in time spent in sedentary activities. From 9 to 10 years onwards, official Physical Education programs start to be mandatory at school and this may reduce this negative trend in increased fat mass, as well as a more sedentary lifestyle of all these children. In a systematic review Shrewsbury and Wardle [19] presented data from cross-sectional research (1990 to 2005) relating SES and adiposity in school-age children from developed countries. They showed consistent negative relationships between SES and adiposity, *i.e.*, highest adiposity was linked to the lowest SES. Further, in adults, a similar review conducted by McLaren [53] also found lower SES associated with larger body size mostly in women from highly developed countries. In the Cariri region children from private schools are also those with higher SES. This condition is more likely to be associated with richer diets as well as higher number of electronic devices and their time use with a consequently higher sedentariness. On the other hand, children from public schools are from a clearly distinct environment. For example, their daily diet is mostly based on natural food such as beans, grains and fish; further, they also walk to and from school, and sometimes help their families in daily chores.

Biological maturation was significantly and positively associated with fat mass accrual in girls and boys. These are expected results since more mature children and adolescents tend to be heavier and taller [18]. For example, in the FELS longitudinal study, Guo *et al.* [54] examined patterns of change in body composition with rates of skeletal maturation from 8 to 20 years of age, and found that total body fat, fat mass percentage and fat free mass consistently increased with increasing rates of maturation. Clear sex-differences in fat mass percentile values and changes were identified in the present study that can be primarily attributed to the action of sex steroid hormones. These sex hormones drive the differences during the pubertal years [55] as well as during childhood [56]. These differences are magnified during the growth spurt and persist into adulthood [57]. In a previous study with Portuguese youth, De Souza *et al.* [27] reported that girls and boys had similar mean BMI values at the age of peak height velocity, and that BMI increased almost linearly with age in both sexes. However, it should be remembered that BMI and fat mass are two distinct phenotypes representing disparent aspects of body composition during growth.

It is important to recognize, at least, two limitations in the present study. First, fat mass was estimated based on regression equations. However, Slaughter equations are largely applied in many studies ranging from Caucasians to Afro-Americans [12,58]. Further, Hussain *et al.* [34] recently showed that the Slaughter equations provided reliable information when compared to DEXA data. When using large samples, it is impractical to use more sophisticated body composition assessment techniques and also that they are very expensive, time-consuming and require specialized technicians to do the evaluations, but we anticipate that BIA may actually be a feasible technique in large samples even though there are some obvious limitations with this technique as well. Second, biological maturation was estimated using the maturity offset suggested by Mirwald *et al.* [37], and their equations have not been validated in Brazilian children and adolescents. Yet, all other methods (sexual maturation and skeletal age) also provide estimates which were never validated with Brazilian children and adolescents. Further, skeletal age assessments would create logistic and financial problems, and the sexual maturation would also create ethical problems. Notwithstanding these limitations, the study has several important strong points. First, the mixed-longitudinal approach is an adequate design to provide enough data for a better understanding of the dynamics fat mass changes as well as its correlates. Second, fat mass reference values and charts are provided for boys and girls using Cole and Green highly efficient statistical method. Third, multilevel modelling is a very flexible and robust statistical procedure to fit fat mass trajectories in both boys and girls, even when data is missing-by-design as is the case of a mixed-longitudinal study. Fourth, the use of a set of time varying predictors as well as fixed predictors allowed for a clearer picture of correlates of fat mass changes. Fifth, the relatively high sample size of our sample.

## 5. Conclusions

In summary, apart from the fact that SES does not act as a moderator in fat mass development, the most important hypotheses were confirmed, namely that Brazilian boys and girls fat mass centile charts show clear sex-differences; also, fat mass longitudinal changes show different trends in boys and girls; further, higher levels of physical fitness seem to act as protective agents in the reduction of BF increases. Thus, we emphasize the importance of physical fitness not only as a putative health marker, but also the need to systematically increase their levels within the school context. We suggest that in the near future researchers will address the question whether biological maturation categories and their links to different physical fitness levels impact %BF development in children and adolescents in different ways.
